# Impact of the COVID-19 Pandemic on Stress, Sleep, and Oral Health in University Students

**DOI:** 10.3389/fpain.2021.744264

**Published:** 2021-10-26

**Authors:** Thaissa Reis do Carmo Cerqueira, Sayene Garcia Batista, Elson Braga de Mello, Marcos Fabio DosSantos, Inger Teixeira de Campos Tuñas

**Affiliations:** ^1^Prosthodontics and Materials Sciences Department, Faculty of Dentistry, Federal University of Rio de Janeiro (UFRJ), Rio de Janeiro, Brazil; ^2^Postgraduate Program in Medicine (Radioloy), Faculty of Medicine, Federal University of Rio de Janeiro (UFRJ), Rio de Janeiro, Brazil; ^3^Department of Social and Preventive Dentistry, Faculty of Dentistry, Federal University of Rio de Janeiro (UFRJ), Rio de Janeiro, Brazil

**Keywords:** COVID-19, psychological distress, bruxism, temporomandibular disorders (TMD), oral health, stress, sleep

## Abstract

**Background:** The COVID-19 pandemic, a disease caused by Sars-CoV-2, has become a worldwide stressor, especially as it represents a new viral infection, which spreads quickly and easily, without prior knowledge about vaccination, and absence, to this moment, of a medication that is totally effective against the disease.

**Objective:** The aim of this observational study was to provide a general evaluation, through a questionnaire applied to students in the health field of the Federal University of Rio de Janeiro, on the psychological impacts and behavioral changes generated by the COVID-19 pandemic on oral health, especially the triggering or exacerbation of bruxism and temporomandibular disorders (TMD).

**Methods:** In order to verify the impacts of the pandemic on the health of UFRJ healthcare students, a non-randomized survey was performed with 370 students.

**Results:** It was found that 72% of the students had their sleep routine altered, 65% had greater difficulty in keeping their spirits up, there was a statistically significant increase in emotional stress, headaches, and daytime teeth clenching.

**Conclusion:** It was possible to conclude that the outbreak of COVID-19 resulted in psychological, physiological and behavioral impacts on students.

## Introduction

In December 2019, an outbreak of pneumonia, widely and rapidly spread, was reported in Wuhan, People's Republic of China. The virus that causes the disease, SARS-CoV-2, is capable of affecting the lower respiratory tract, and has been identified as a new strain of Coronavirus. The virus has spread to other countries and the World Health Organization (WHO) declared, in January 2020, a public health emergency of international concern. In early February of the same year, it was announced that the disease caused by the new coronavirus would be called COVID-19 (“CO” for corona, “VI” for viruses and “D” for disease). On March 11, 2020, the WHO decreed that the outbreak characterized a pandemic (1–6).

Global attention has turned to protocols that could reduce transmission and the impacts of the COVID-19 pandemic. In this context, the WHO recommended preventive measures such as frequent hand washing and social distancing to reduce the contagion curve. The terms social distancing, quarantine and isolation started to be used frequently by the population (4, 5, 7). Social distancing seeks to limit social interaction in order to reduce the spread of given disease, through avoiding crowding; quarantine refers to the separation and restriction of movement of people who have been potentially exposed to a contagious disease, in order to check whether they will manifest symptoms, and isolation characterizes the separation of people diagnosed with a contagious disease from those who are not infected (1, 5–7). Such measures to contain the spread of the pandemic can affect the psychological state of the population (8–13).

All these factors, which are notably stressors, led to a rapid change in social habits due to the imminent risk inherent in the pathological condition. In this context, it would be plausible to consider an increase in cases of temporomandibular disorders (TMD) and bruxism, as they are psychosomatic oral diseases closely related to psychosocial and environmental factors (14), in addition to possible excessive use of alcohol and tobacco, and antidepressant medications (15, 16). In psychologically vulnerable individuals, psychosocial aspects can play an important role in maintaining orofacial pain. Stress is a condition capable of boosting the occurrence of TMD and, additionally, high levels of emotional stress can increase the muscle tone of the head and neck as well as the levels of parafunctional muscle activity, represented by bruxism or teeth clenching (17, 18). Furthermore, the increase in muscle tone caused by stress can induce muscle pain (1, 5–7, 19).

The aim of this study was to evaluate the psychological impact of the COVID-19 pandemic on oral health, with special attention to the possible triggering or exacerbation of bruxism and temporomandibular disorders (TMD).

## Materials and Methods

In this observational study an online 24-question questionnaire was applied to healthcare students of the Federal University of Rio de Janeiro, Rio de Janeiro, regarding the effects of the psychological impact and behavioral changes generated by the COVID-19 pandemic on oral health, with special attention to the possible triggering or exacerbation of bruxism and temporomandibular disorders (TMD).

A non-randomized survey was conducted through an online questionnaire structured on the GOOGLE FORMS platform, created exclusively for this experiment. Undergraduate students of Nursing, Physiotherapy, Medicine and Dentistry at the Federal University of Rio de Janeiro were the inclusion criteria to participate in the research and answer closed questions on the topic. Students from these areas were chosen because they are the ones who are most involved in the practice concerning the pandemic. In addition to the stress that is common to all, these students are living with the possibility of contamination and closely following the suffering of patients, aside from having been affected in terms of their academic calendars and deadlines for completing their graduation. This was an observational study. STROBE guidelines for observational studies have been followed. STROBE checklist for observational studies has also been completed (Supplementary Material 1). Students who: (a) did not meet the inclusion criteria; (b) submitted their forms after the deadline and (c) refused to accept the Informed Consent Form, were excluded from the survey. Data were analyzed using McNemar and Chi-square tests, with BioStat software, version 5.3. The questionnaire was sent through the internet (instant messaging application) from a list acquired from the representative students of each course. Students had access to the form between 11.24.2020 and 02.09.2021. Data were collected after approval by the Ethics in Research Committee of the Clementino Fraga Filho Hospital - Federal University of Rio de Janeiro/HUCFF - UFRJ, through Opinion 4.413,268.

To determine the number of respondents, a sample calculation for survey studies was performed (Commentto®) using as parameters the population of 2,450 undergraduate students from the schools of Nursing, Physiotherapy, Medicine and Dentistry at UFRJ, homogeneously distributed, with a sampling error of 5% and a 99% confidence level, which resulted in 363, with responses from 370 students. Effect size was not calculated.

## Results

The study population consisted of 370 students: 97 Nursing students, 94 Dentistry, 94 Medicine and 85 Physiotherapy students. Students from different semesters answered the questionnaire, but the third semester was the most representative with 19.5% (72) of the responses. Female students represented 80.8% of respondents. The most prevalent ages were between 20 and 22 years old, which corresponded to 51.1% of the students.

**Figure 1:**
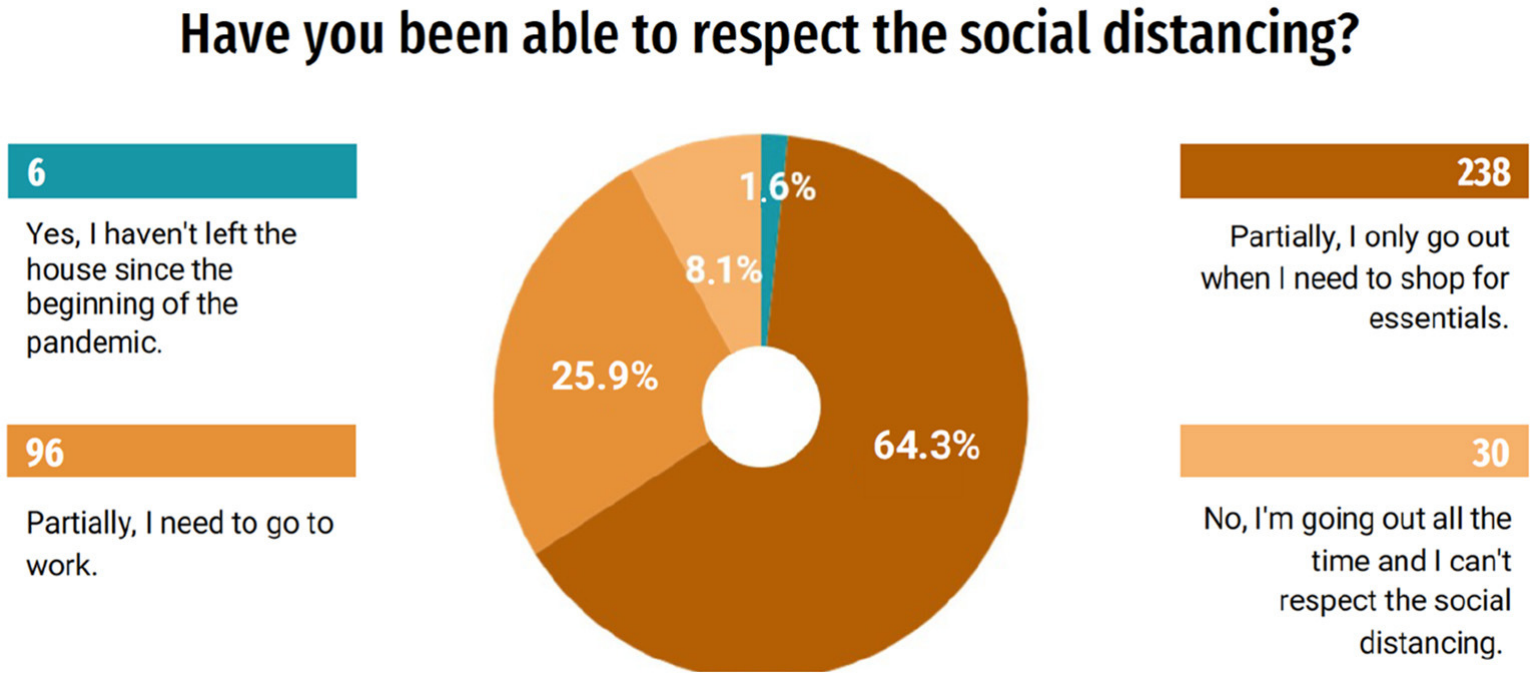
Respect for the recommended social distancing.

According to the questionnaire responses, 95.9% (355) of the students expressed concern about COVID-19 and are using preventive measures at home, as recommended by health authorities, such as frequent hand hygiene and use 70% alcohol for cleaning surfaces. Despite the concern about the disease, only 1.6% (6) of the students have not left home since the beginning of the pandemic. A significant number of students, 64.3% (238), say they can partially respect social distancing, by going out only when it is necessary to shop for essential items, while 25.9% (96) need to go out to work and 8.1% (30) of the students are always leaving and are unable to respect social distancing (Figure 1). The questionnaire also revealed that only 18.1% (67) of the students had been interns in hospitals or clinics during the pandemic so far.

**Figure 2:**
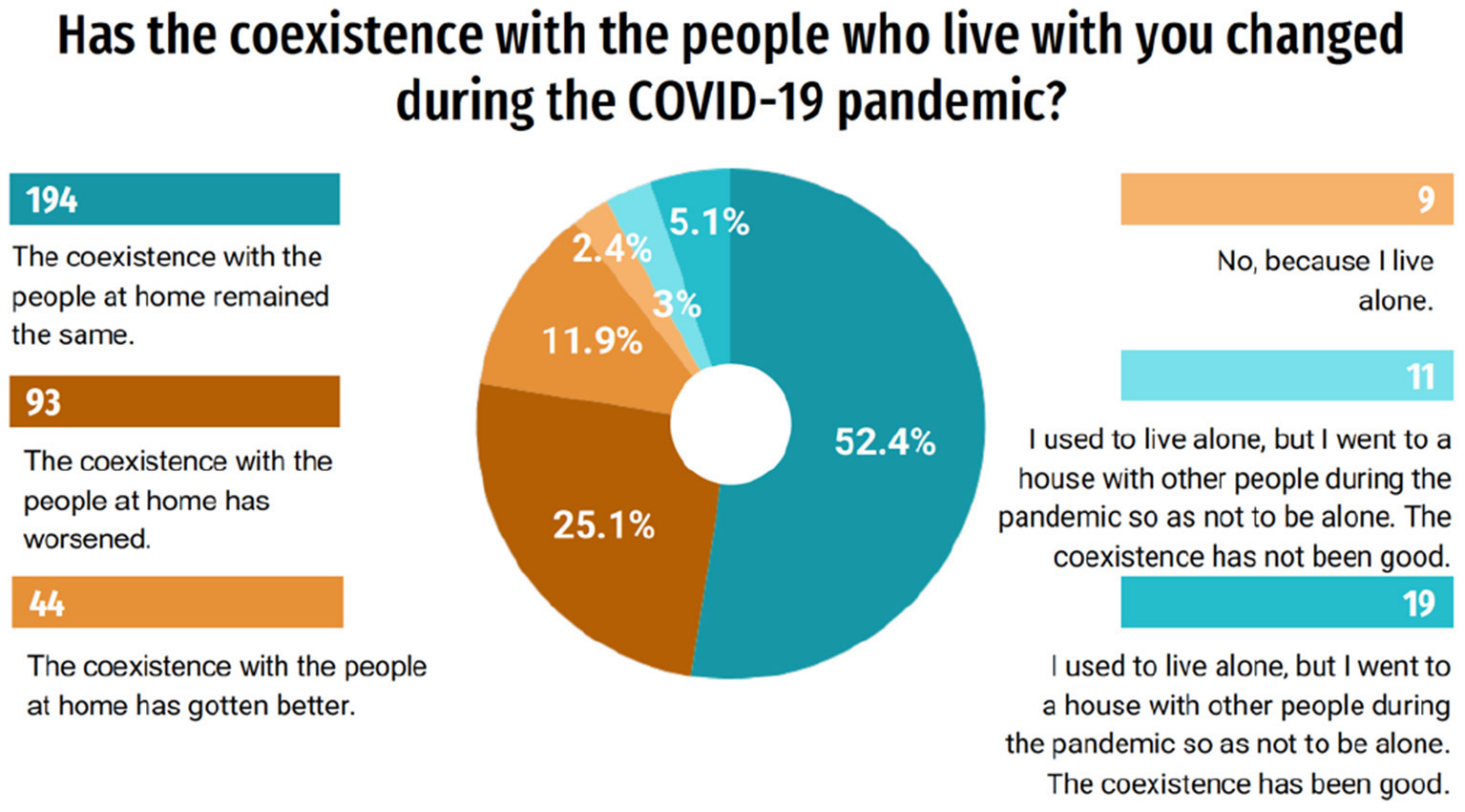
Coexistence with the people who live with the student during the pandemic.

**Figure 3:**
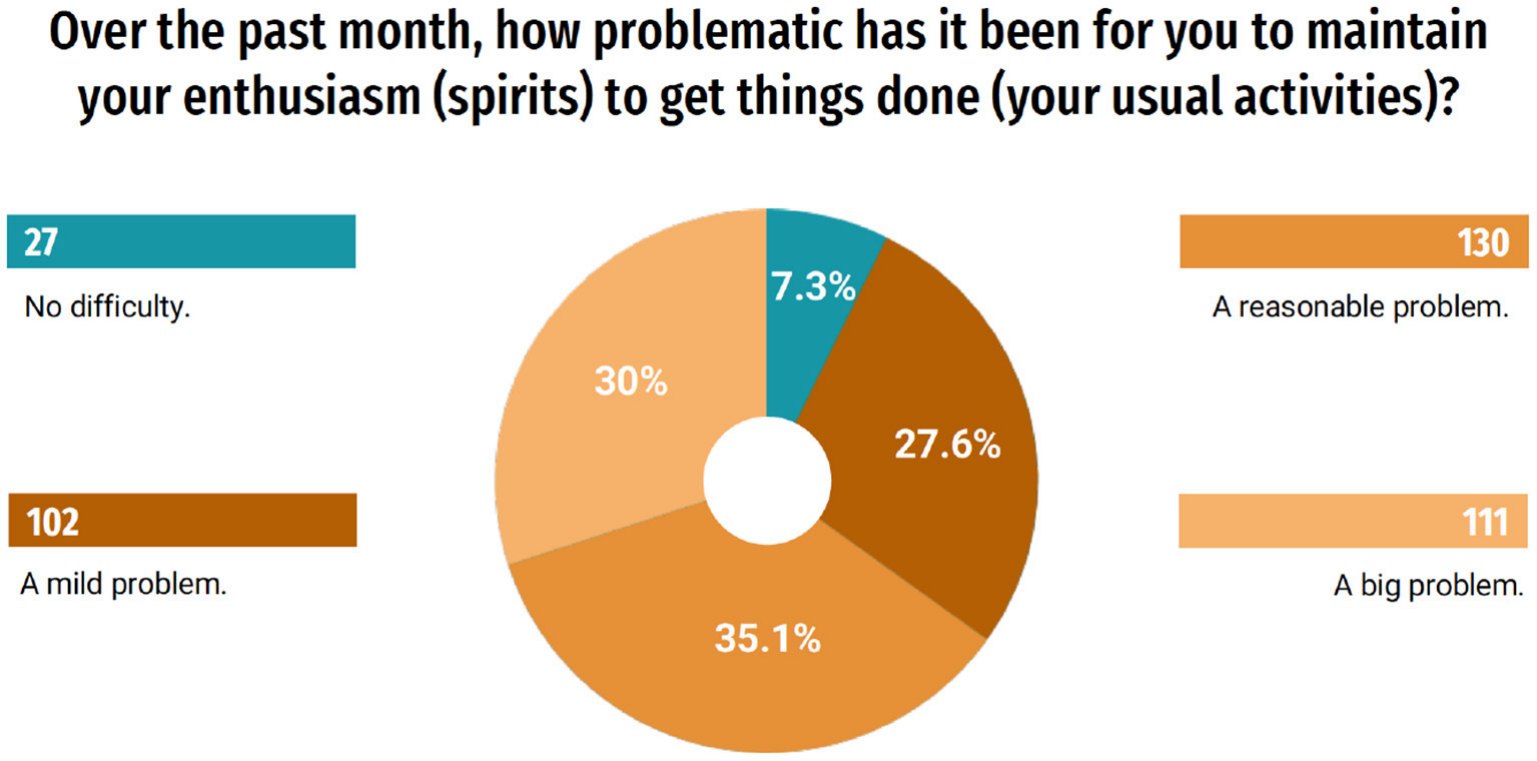
Maintenance of enthusiasm for usual activities.

When asked about whether the cohabitation with the people who live with the student changed during the pandemic, 52.4% responded that it remained the same, however, 25.1% (93) of the students said that the coexistence worsened (Figure 2). Moreover, according to the answers about how problematic it was to maintain enthusiasm for carrying out usual activities during the last month, 30% (111) considered it a great problem (Figure 3).

The period of the COVID-19 pandemic and the need for social restrictions to contain the virus resulted in a global financial crisis. Therefore, students were also asked about the economic factors in the pandemic. The income of 52.2% (193) of the students or people living with them was reduced during the COVID-19 pandemic. And most undergraduates feel somehow pressured to help with household expenses due to the pandemic (54.3%).

**Figure 4:**
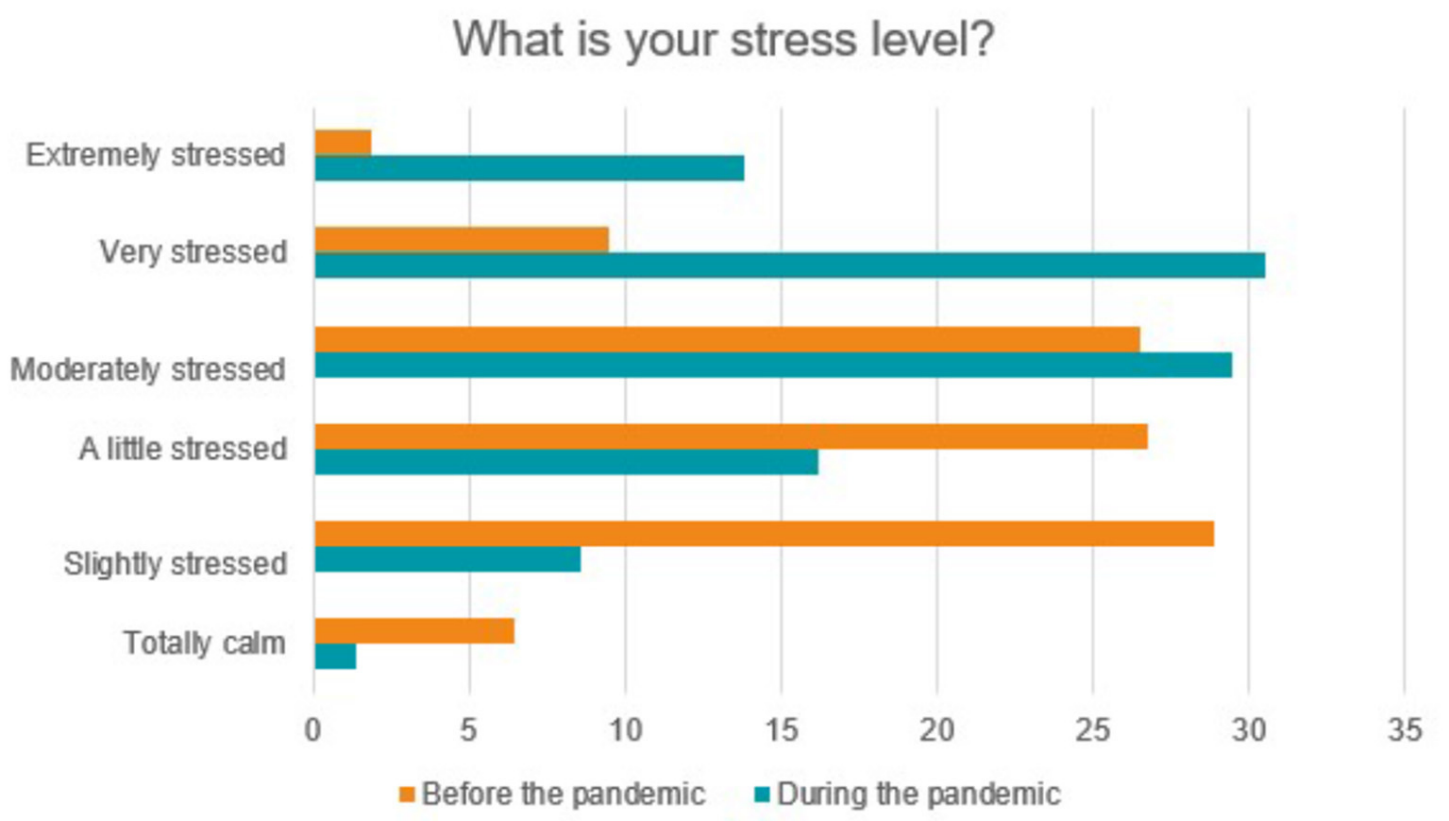
Stress level before and during the pandemic.

When questioned about the level of stress before and after the pandemic, 9.5% (35) of the respondents considered themselves very stressed before the pandemic and 30.5% (113) of the students during the pandemic (Figure 4). Comparing the 6 categories of stress levels used in this study, before and during the pandemic, *p* < 0.0001 was found, that is, a highly significant difference (Chi-square). It was also questioned whether there was a change in the consumption of alcoholic beverages during the pandemic, and it was found that 33% (122) observed a reduction in frequency and 10% (37) an increase in consumption.

**Figure 5:**
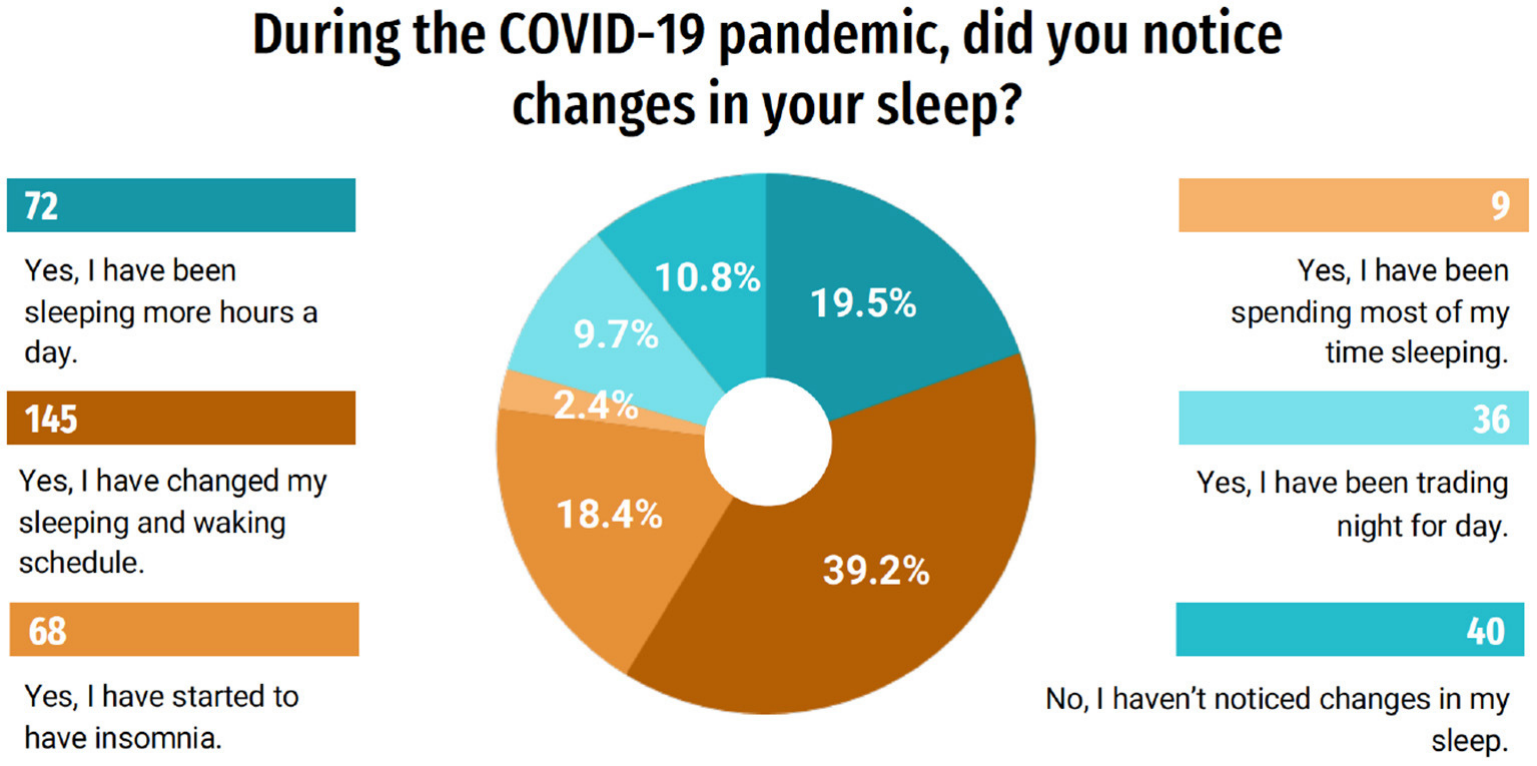
Changes in sleep routine.

**Figure 6:**
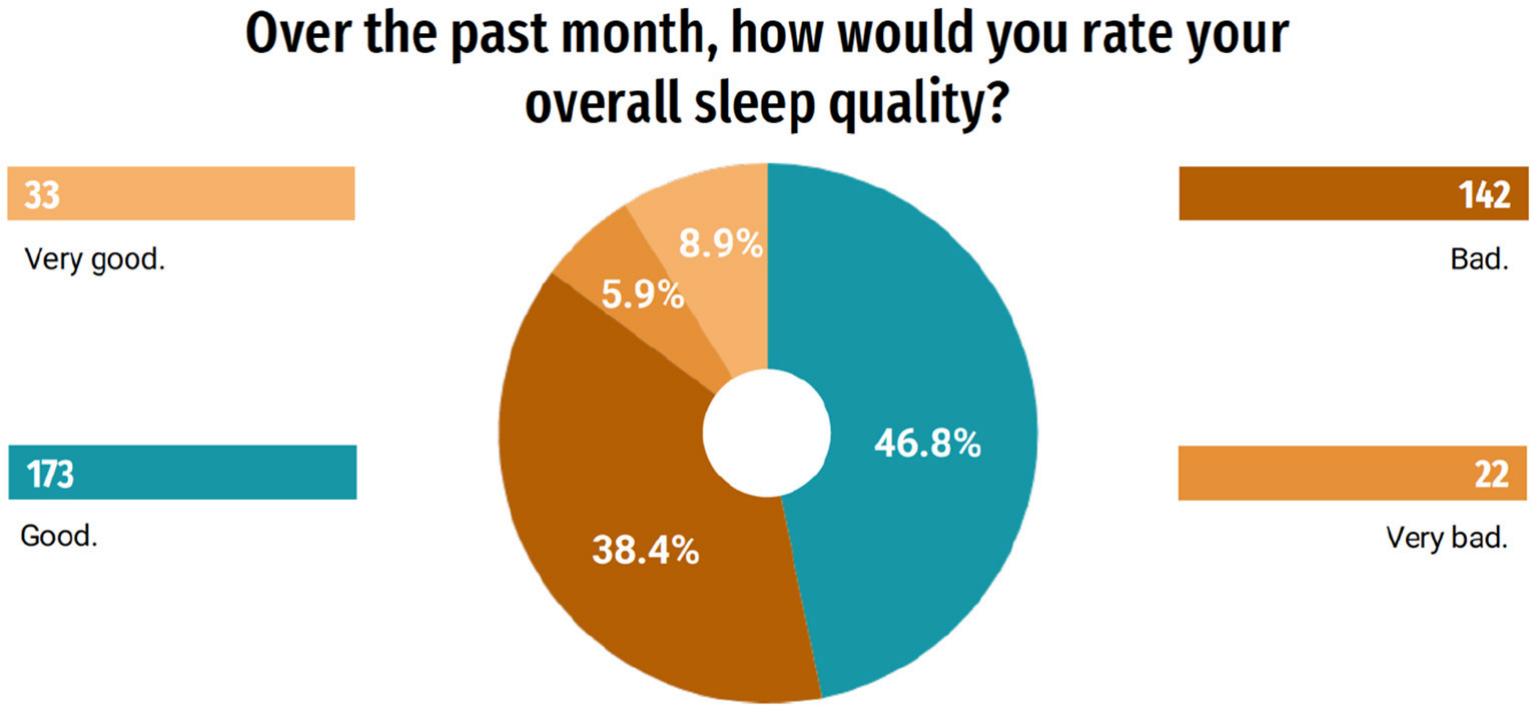
Quality of sleep.

**Figure 7:**
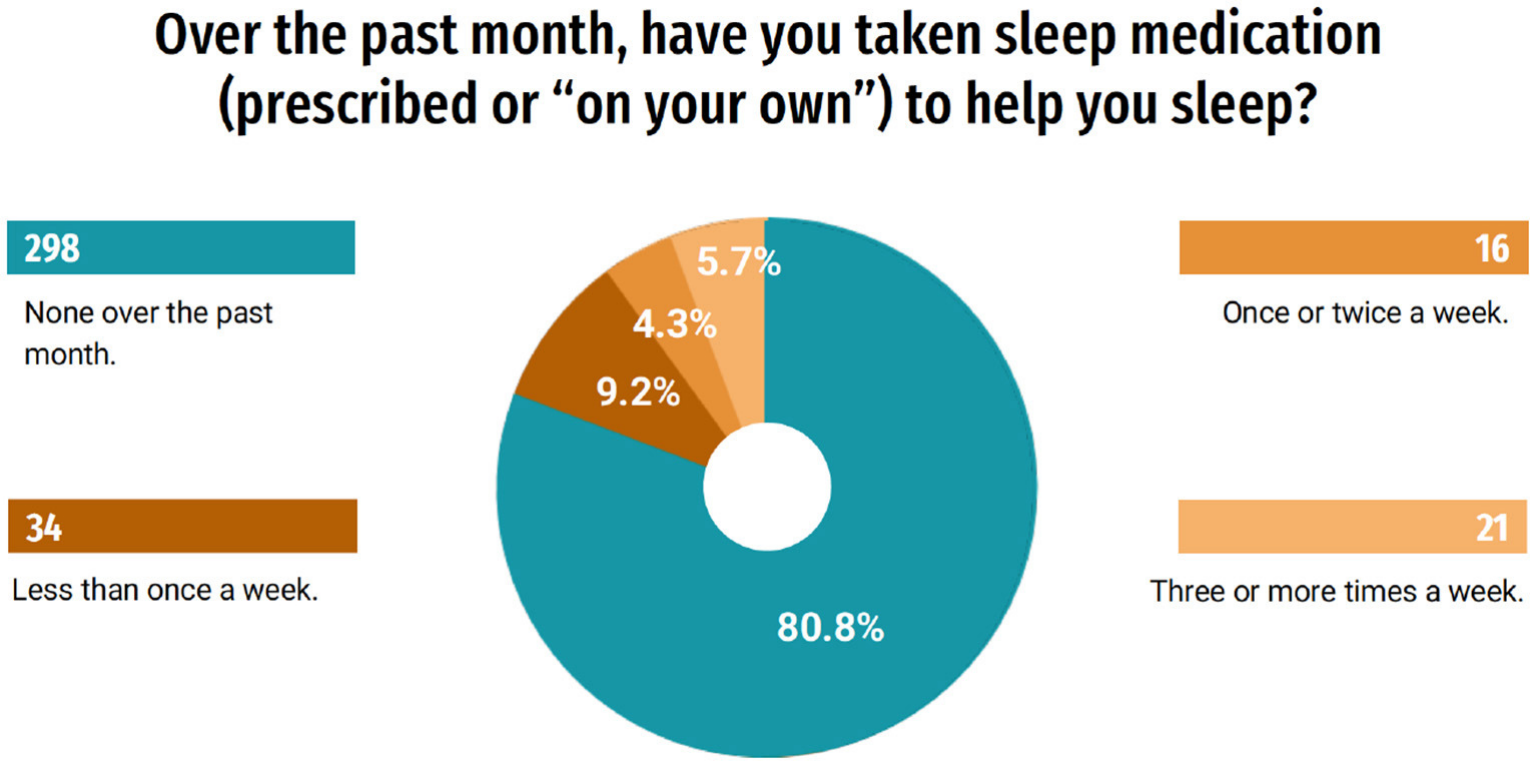
Medication to help going to sleep.

**Figure 8:**
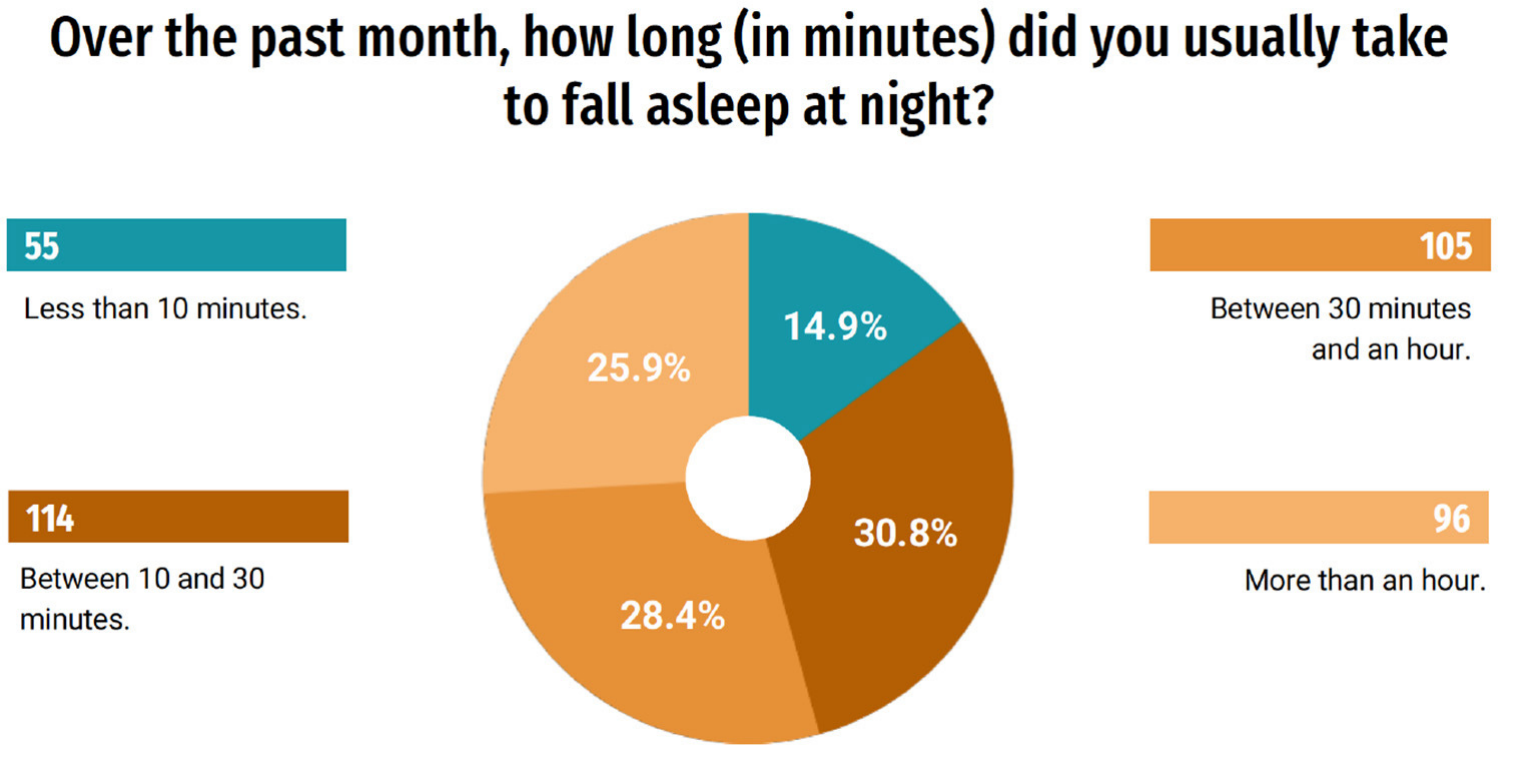
The time it takes to sleep at night.

In order to better understand the influence of the pandemic on the quality of life of this group, questions related to sleep evaluation were asked. Of the students, 39.2% (145) responded that they changed their sleeping and waking schedules and 19.5% (72) responded that they have slept more hours a day, and 18.4% (68) began to have insomnia. Only 10.8% (40) of students responded that they did not notice changes in sleep (Figure 5). When questioned about their quality of sleep, 55.7% (206) rated it, in general, as very good or good; on the other hand, 44.3% (164) classified it as bad or very bad (Figure 6). A part of the students, 19.2% (71), indicated that during the last month they used some medication to help them sleep (Figure 7). When answering about the time they usually took to sleep at night, also during the last month, 25.9% (96) of the students reported that it took more than 1 h (Figure 8).

**Figure 9:**
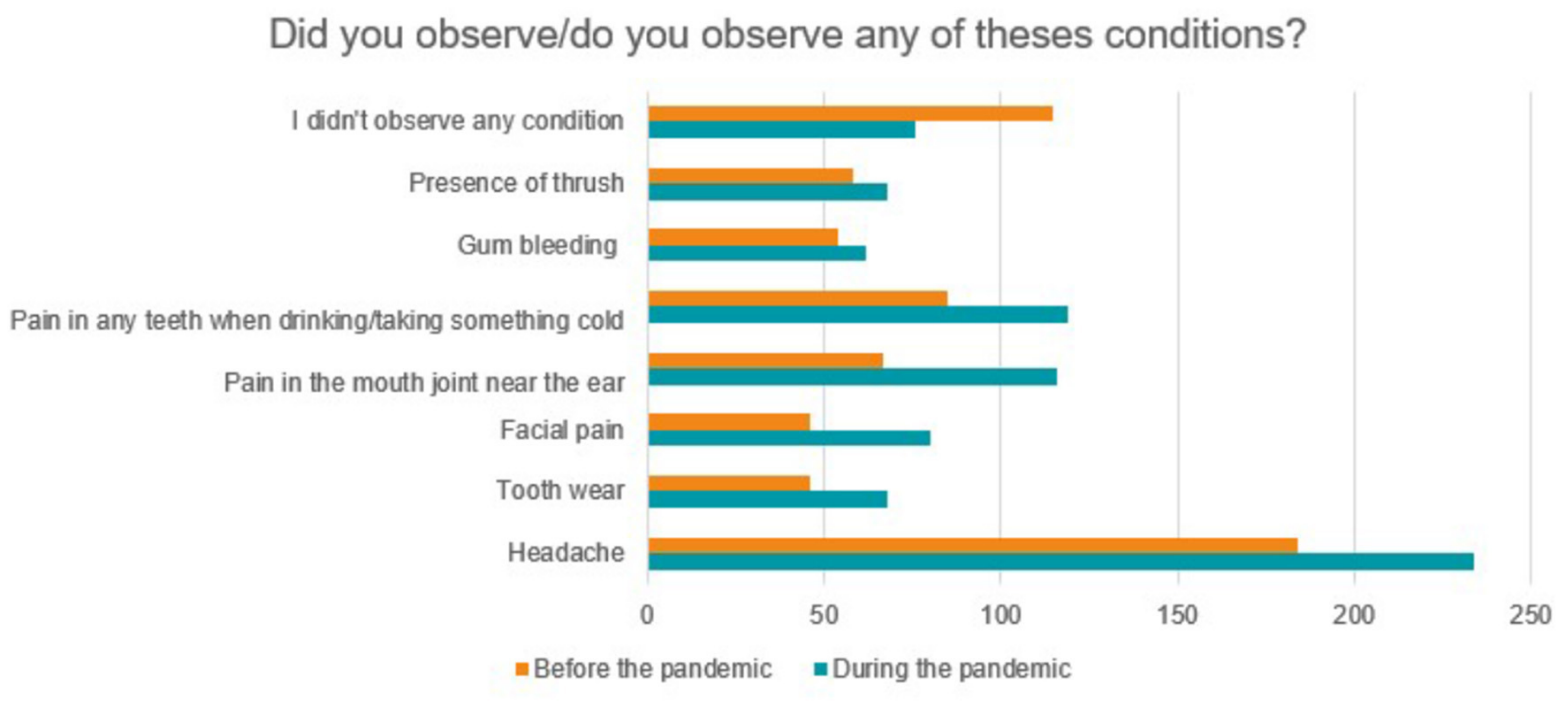
Conditions observed before and during the pandemic.

**Figure 10:**
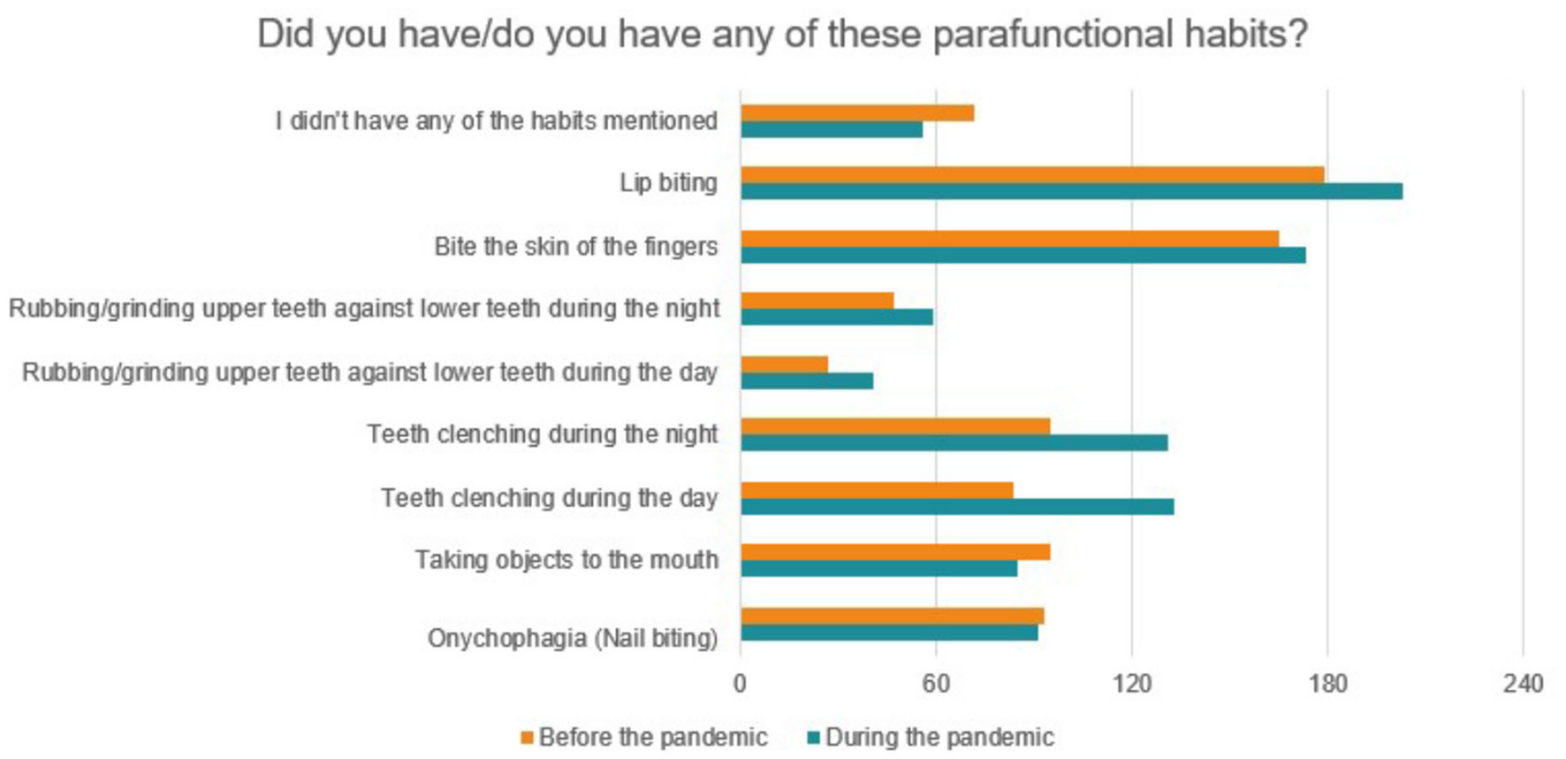
Parafunctional habits observed before and during the pandemic.

The occurrence of several orofacial alterations observed before and after the pandemic was also investigated, as shown in Figure 9. In general, there was an increase in the referred conditions during the pandemic, however, only for headache there was a statistically significant difference (*p* = 0.0063). Among parafanctional habits, a statistically significant increase was found only for daytime teeth clenching (*p* = 0.0485), based on McNemar's non-parametric test, of two related samples (Figure 10). Eighty-four students responded that they performed daytime clenching before the pandemic and this number increased to 133 students during the pandemic, that is, 49 students (13.24%) started to perform this parafunctional habit.

## Discussion

The questionnaire was made available in November 2020, almost a year after the first case of COVID-19 in Wuhan, China. Despite the time elapsed, the world scenario still requires strategies to fight the virus. By the time respondents answered the questionnaires, the first vaccines that passed the testing phases were announced in different laboratories around the world. Nevertheless, social activities in the country required social distancing and remained restricted, while still awaiting the availability of these vaccines. As in other education institutions, at the University of the students in this research, the activities started to have a predominantly remote character, with permission to function only for internships such as clinical activities (1–6).

Social distancing has completely changed the population's daily routine, creating some challenges in the lives of many families. People who live in the same way at home have never been as close together over time as in the COVID-19 pandemic. Commercial activities are now carried out inside the houses of employees of various companies; and students began to conduct their tasks at home, through remote learning (4, 5, 7). In the present study, 25.1% (93) of the students revealed that the coexistence at home worsened. This may be related to the need to share and manage the same space, as well as the same equipment to perform different individual activities. The isolation of families at home, imposed by the new coronavirus, challenged the population to rediscover new ways of interacting and living with each other.

The efforts to contain the COVID-19 virus have resulted in a worldwide economic recession. The sudden drop in demand for goods and services and the high unemployment rate have aggravated the complexity of the situation. The present study also showed the effects of COVID-19 on the students' economy. The income of 52.2% (193) of students or people living with them was reduced during the COVID-19 pandemic. In addition, 54.3% of undergraduates responded that they feel somehow pressured to help with household expenses due to the pandemic. Inevitably, the post-pandemic scenario will require changes and strategies for restructuring and economic recovery (8, 9, 11, 19, 20).

Even with the imposition of various measures to contain the spread of COVID-19 and minimize the number of deaths, the pandemic situation has caused thousands of deaths across the planet, challenged professionals, and collapsed the health systems of many countries. When facing the uncertainties of the control of the pandemic, people are more worried, stressed and find it more difficult to keep their spirits up. This can lead to alterations in physiological activities of the body, such as changes in the sleep routine. Among the students of the study, 72% observed changes in their sleep routine. Sleep plays a fundamental role in emotional regulation. Thus, sleep disorders can have direct consequences on emotional functioning, weakening various systems and functions such as the immune system, learning and memory (10, 20). The current study also revealed that 18.4% (68) of students began to experience sleep restriction. It is known that individuals who are sensitive to sleep disruption related to stress are more likely to develop chronic changes in their sleep routine (20). It is noteworthy that sleep disorders occur in approximately one third of TMD cases and seem to promote an increase in pain intensity over time (21). In fact, intensification of bruxism together with TMD signs and symptoms, putatively associated with higher levels of orofacial pain has also been reported in both Polish and Israeli populations, using on-line surveys (22).

The impacts of the pandemic can be observed in numerous sectors. Health crises arising from newly discovered diseases involve the inexperience of health professionals and create instabilities. Interruptions in everyday life such as unemployment, the loss of relatives, suspension of academic activities, reduction of social interaction, immersion in the news about the outbreak followed by rumors and “fake news,” cause discomfort, anxiety and stress (8, 9, 11, 19, 20). In addition to affecting the physical health of the population, COVID-19 has severely influenced everyone's psychological health (2, 23, 24). Thus, mental disorders related to COVID-19 are the result of biological, social and psychological factors (25).

In this situation, the body is in a state of alert and, as a result, responses to stress occur (10). The possibility of illness itself is a stressor, and when people feel that their lives are in imminent danger, a series of internal psychological reactions manifest themselves; fear, anxiety, sadness, depression, irritability, hypersensitivity, anguish, sleep disturbances, suicide and psychophysiological reactions such as fatigue, pain, palpitations, chest tightness, muscle tension and decreased appetite are possible (10, 26, 27).

After the occurrence of an emergency, certain stress symptoms may appear as early as 1 day after exposure to the event, and the psychological impact on some people may last for several years (28). Psychological well-being and sleep are affected by many sociocultural factors, such as economic burden, family support and social support (27, 29, 30). In the present study, stress and daytime clenching (awake bruxism) increased significantly among healthcare undergraduate students of UFRJ, a group that is mostly composed of young people aged from 20 to 25 years old. Thus, one can relate the pandemic scenario, along with greater idleness and lack of social interaction as stressful factors, to the increase in daytime clenching (31).

According to the International Consensus of 2018, awake bruxism is a masticatory muscle activity performed when the individual is awake, characterized by repetitive or sustained contact of the teeth and/or forced maintenance of the mandibular position or movement (32). In terms of clinical consequences, bruxism can be classified in three ways. It can be a harmless behavior or even a protective factor (e.g., when preventing collapse or restoring the upper airways patency during sleep). However, there is also the possibility that bruxism is a risk factor for negative repercussions on oral health, such as intense masticatory muscle pain or pain in the temporomandibular joints, extreme mechanical wear of the teeth and prosthetic complications (32). Noteworthy, the results of a systematic review indicated that the current scientific literature does not support a direct and linear causal relationship between bruxism and musculoskeletal signs/symptoms. Instead, it pointed toward a far more complex relationship that depends on the presence of other risk factors. The same study highlighted the importance of increasing the focus on non-painful musculoskeletal symptoms in future research studies (33). Interestingly, another study showed no positive correlation between the amount of self-reported bruxism and pain intensity related to TMD. Moreover, though the amount of awake bruxism initially exhibited a positive correlation with TMD-related pain, such correlation was not found when the adopted model was controlled for depression (34). Therefore, the concept that more bruxism is necessarily associated with more TMD-related pain could not be confirmed in that study and must be reexamined.

Awake bruxism has been associated with psychosocial factors such as anxiety and stress. Muscle contraction can be part of the defense behavior and fight or flight responses, associated with stressful episodes (1, 15, 35–38). Under stress-free conditions, regions of the prefrontal cortex (PFC) regulate behavior, thinking, and emotion, including inhibiting inappropriate motor responses. However, under stressful conditions such as the pandemic, the amygdala activates pathways in the hypothalamus and brainstem and impairs the regulation of the PFC (39, 40). Therefore, muscle contraction in awake bruxism may be part of the defense behavior associated with anxiety and stress (1). A systematic review of the literature classified emotional stress as a very high risk factor for awake bruxism (36), which may increase the chance of this type of bruxism in patients with high levels of stress by up to six times (41).

It is worth mentioning that, along with the emotional responses resulting from the pandemic and confinement, there may be an increase in unhealthy behaviors, such as excessive use of alcohol and tobacco, as a form of distraction or as a consequence of stress, anxiety or depressive symptoms that are being experienced (42–44). In the present study, a reduction in the frequency of consumption of alcoholic beverages was verified, possibly as a result of the reduction in the income of undergraduates, as well as the social restrictions imposed by the pandemic (45). The global situation resulting from the COVID-19 pandemic and its consequences, which will still persist in the post-pandemic context, will certainly cause psychosocial changes in the population (31). These changes can aggravate or trigger stomatognathic problems. Post-pandemic signs can be similar to post-traumatic stress syndrome. The presentation or exacerbation of symptoms of chronic orofacial pain will possibly be a reality for many people in post-traumatic circumstances (17, 28, 38, 46–48).

Therefore, dentists need to be aware of the signs and symptoms resulting from the current moment in order to diagnose a possible awake bruxism. It is the professional's job to explain that teeth clenching is an involuntary behavior, hence, it is necessary to guide their patients about functional and parafunctional contacts so that they can try to change their habits (49). Functional occlusal contact mainly occurs during swallowing and in ephemeral moments of chewing and speaking, lasting 18 min on average at the end of a 24-h cycle (50). Thus, the patient must understand that it is not functional to keep the teeth together while the jaw is resting. Non-functional occlusal contacts lead to increased muscle activity (mainly masseter and temporal), causing hypertonia and consequent myalgia, one of the factors of orofacial pain (34, 51). That being said, the importance of behavioral advice to control awake bruxism and mitigate its consequences is highlighted. To this end, it is recommended to get into the habit of keeping the teeth unclenched and with the lips sealed in the resting position. Apps that issue alerts reminding you to unclench your teeth are a good strategy, as does the “Desencoste” app, available for Android and IOS for free (52).

Although the present work provided interesting and novel data related to psychological effects of the COVID-19 pandemic on healthcare students, especially their level of bruxism, some limitations of the study should be considered. For instance, this was a non-randomized study (e.g., the distribution of the questionnaire was not randomized). Thus, it is possible to hypothesize that only students who were most affected by the COVID-19 pandemic took part in the survey. Furthermore, only 15% of eligible students responded the applied questionnaire and a higher prevalence of the responders were female (81%). Although the relevance of gender as an important factor to the development of TMD has been demonstrated (a two times greater risk of women to develop TMD when compared to men) (53) a gender bias in the current study cannot be excluded. In fact, the results of the present study must be interpreted cautiously.

Noteworthy, the results of this study were based on a survey. Therefore, data from the gold standard methods used for diagnosis of bruxism such as polygraphic and audio-video recording (50, 54) could not be assessed. Other studies that analyzed the effects of the COVID-19 pandemic on bruxism and TMD through on-line surveys have the same type of limitation. Nevertheless, all bring important information (22). Such information must be confirmed expanded in further studies. In addition, no validated tools were used to assess the levels of stress and the quality of sleep. As a consequence, a responder recall bias cannot be discarded either, when considering the pre-pandemic bruxism and other health conditions.

From the point of view of the social role, the dentist performs not only the role of specialist for professional guidance in Dentistry, but also the role of assisting in counseling, suggesting a multidisciplinary treatment to patients, including medical and psychological (55, 56). Awake teeth clenching with non-restorative sleep, even without the presence of sleep bruxism, can lead to more pain in the morning (21). Sleep is a physiological need for nutrition and metabolism maintenance, and, consequently, sleep hygiene must be explained by the professionals involved, and taught to the patients (10, 21). Another important point is to avoid postural behaviors during sleep that can overload the joints and muscles of the face region (52). In view of the pandemic scenario, it is essential to consider the relationship between the psychological status of patients and oral diseases.

## Conclusion

From the responses obtained from the questionnaires regarding the respondents, it was possible to conclude that there is a great concern with COVID-19 and with the reduction of family income during the pandemic period. Moreover, they feel pressured to help with household expenses. Students are also struggling to keep their spirits up, have an altered sleep routine and a significant increase in the level of emotional stress and in the occurrence of daytime clenching, which are triggering factors for TMD. Furthermore, headaches, which may be related to TMD, had a significant increase. The COVID-19 outbreak resulted in psychological, physiological and behavioral impacts on students.

## Author Contributions

TC, SB, EM, and IT developed and designed the study. TC, SB, EM, MD, and IT analyzed the data and drafted the manuscript. All authors had full access to all the data in the study.

## Funding

MD received fellowships from the following Brazilian funding agencies: Conselho Nacional de Desenvolvimento Científico e Tecnológico (CNPq) - PQ2 (09/2020) and Fundação de Amparo à Pesquisa do Rio de Janeiro (FAPERJ) - JCNE 2018.

## Conflict of Interest

The authors declare that the research was conducted in the absence of any commercial or financial relationships that could be construed as a potential conflict of interest.

## Publisher's Note

All claims expressed in this article are solely those of the authors and do not necessarily represent those of their affiliated organizations, or those of the publisher, the editors and the reviewers. Any product that may be evaluated in this article, or claim that may be made by its manufacturer, is not guaranteed or endorsed by the publisher.
